# Targeting PCNA/AR interaction inhibits AR-mediated signaling in castration resistant prostate cancer cells

**DOI:** 10.18632/oncotarget.28722

**Published:** 2025-05-20

**Authors:** Shan Lu, Zhongyun Dong

**Affiliations:** ^1^Department of Internal Medicine, University of Cincinnati College of Medicine, Cincinnati, OH 45267, USA

**Keywords:** PCNA, androgen receptor, PCNA inhibitors, AR splicing variants, CRPC

## Abstract

We previously showed that proliferating cell nuclear antigen (PCNA) interacts with androgen receptor (AR) through a PIP-box (PIP-box4) at the N-terminus of AR and regulates AR activity. In this study, we further investigated PCNA/AR interaction. We identified a second PIP-box (PIP-box592) in the DNA binding domain of AR and found that dihydrotestosterone enhances the binding of full-length AR (AR-FL) but not a constitutively active variant (AR-V7) to PCNA. Treatment with R9-AR-PIP, a PIP-box4-mimicking small peptide, inhibits the PCNA/AR interaction, AR occupancy at the androgen response element (ARE) in *PSA* and *p21* genes, and expression of AR target genes, and induces cytotoxicity in AR-positive castration-resistant prostate cancer (CRPC) cells. R9-AR-PIP also significantly inhibits transcriptional activity of AR-FL upon dihydrotestosterone stimulation and the constitutive activity of AR-V7. Moreover, R9-AR-PIP and PCNA-I1S, a small molecule PCNA inhibitor, inhibit the ARE occupancy by AR-FL and AR-Vs in *CCNA2* gene that encodes cyclin A2 and cyclin A2 expression. Finally, we found that cyclin A2 is overexpressed in all CRPC cells examined, suggesting that it may contribute to the development of CRPC. These data indicate that targeting PCNA/AR interaction inhibits both AR-FL- and AR-Vs-mediated signaling and implicates it could be a novel therapeutic strategy against CRPC.

## INTRODUCTION

Almost all advanced prostate cancers eventually progress to castration resistant prostate cancers (CRPC) upon androgen deprivation therapy (e.g., bicalutamide) [1–4]. CRPC overexpress the full-length androgen receptor (AR-FL) and/or the constitutively active AR splicing variants (AR-Vs) [2, 5–10]. AR-V7 and ARv567es are two predominant AR-Vs, which have lost all or part of the ligand binding domain. The constitutively active AR-Vs function as AR-FL in an androgen independent manner in binding to the androgen response element (ARE) and interact with coregulators via the N-terminal domain [11]. Treatment of CRPC with androgen receptor pathway inhibitor, such as enzalutamide by blocking the activation of AR-FL and abiraterone by inhibition of androgen synthesis, prolongs the patient survival by 4–5 months [12, 13]. Microtubule inhibitors docetaxel and cabazitaxel are chemotherapy drugs to prolong the survival by 2–3 months [12]. For metastatic CRPC, phase III clinical trials are examining the triplet therapy regimens, such as androgen deprivation therapy, docetaxel, and androgen receptor pathway inhibitor [14, 15]. Currently, there is no effective therapy for CRPC.

Proliferating cell nuclear antigen (PCNA) is a multifunctional protein essential for DNA replication and repair as well as cell growth and survival [16]. PCNA is a ring-shaped homotrimer located mainly in the nucleoplasm and must be linearized or monomerized for re-localization to chromatin, cytoplasm, or cell membrane to play its function [17–23]. It functions through interaction with partner proteins containing the PCNA-interacting protein-box (PIP-box), AlkB homologue 2 PCNA-interacting motif (APIM), and/or other motifs [19, 20, 24–26]. PCNA is preferentially overexpressed in all tumors [20]. In prostate cancer, we and others have shown that PCNA overexpression is associated with advanced Gleason scores and pathological stages [27–31], and has significant prognostic value for disease-free survival [28–30]. PCNA overexpression is also a biomarker for diagnosis and prognosis in various types of cancer [32, 33]. The small peptides ATX-101 mimicking the APIM completed a phase 1 clinical trial in patients with solid tumors [34]. It is currently in a Phase 1b/2a clinical trial to investigate its combination effects with platinum-based chemotherapy in patients with platinum sensitive ovarian cancer (NCT04814875) [34]. We discovered a series of small molecule PCNA inhibitors that bind to PCNA trimers at the interfaces of two monomers, stabilize the trimer structure, and interfere with PCNA re-localization to the nucleus and chromatin association for function [35]. PCNA inhibitors PCNA-I1 and PCNA-I1S induce DNA damage, inhibit DNA repair, and enhance DNA damage induced by other agents [36–38].

**Figure 1:**
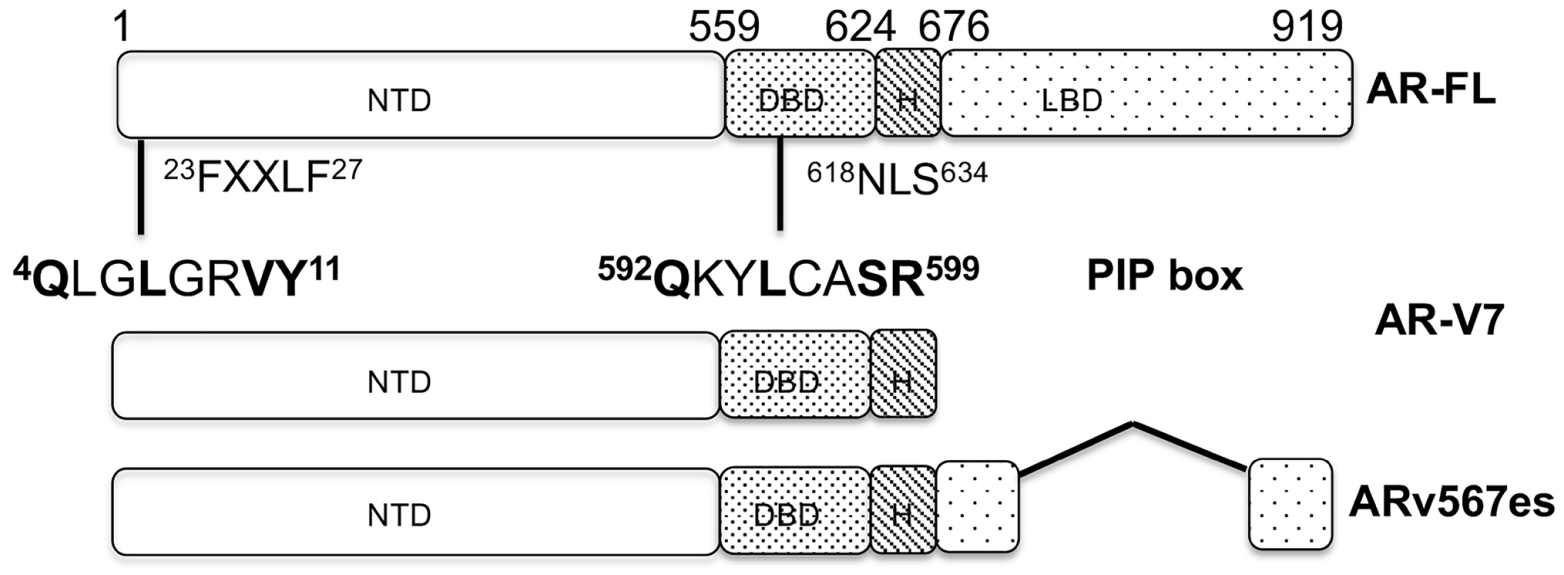
The functional domains of AR-FL and AR-Vs. PIP box4 and PIP box592 are indicated. Abbreviations: NLS: nuclear localization signal; NTD: N-terminal domain; DBD: DNA binding domain; LBD: ligand binding domain (LBD).

We previously investigated PCNA/AR interaction, identified a PCNA interacting protein-box (PIP-box4, ^4^QLGLGRVY^11^) at the N-terminus of AR, and developed a small peptide inhibitor R9-AR-PIP mimicking the PIP-box4 (Figure 1) [39]. PCNA complexes with AR-FL, AR-V7, and ARv567es via the PIP-box and enhances AR transcriptional activity and expression of AR target genes. Blocking PCNA/AR interaction by small molecule PIP-box inhibitor T2AA or R9-AR-PIP inhibits AR activity and expression of AR target genes. R9-AR-PIP also significantly inhibits the growth of AR-positive but not AR-negative prostate cancer cells [39]. The AR activity is also attenuated by PCNA-I1S. The current study identified a second PIP-box592 (^592^QKYLCASR^599^) at the DNA binding domain of AR. Furthermore, PIP-boxes in AR-V7 and ARv567es significantly contribute to their constitutively active activities. Targeting PCNA/AR interaction using PCNA inhibitors compromises AR signaling and induces cytotoxicity in several lines of CRPC cells. Our studies implicate that targeting PCNA/AR interaction could be a novel therapeutic strategy against CRPC.

## RESULTS

### The role of the PIP-box in PCNA/AR interaction

**Figure 2:**
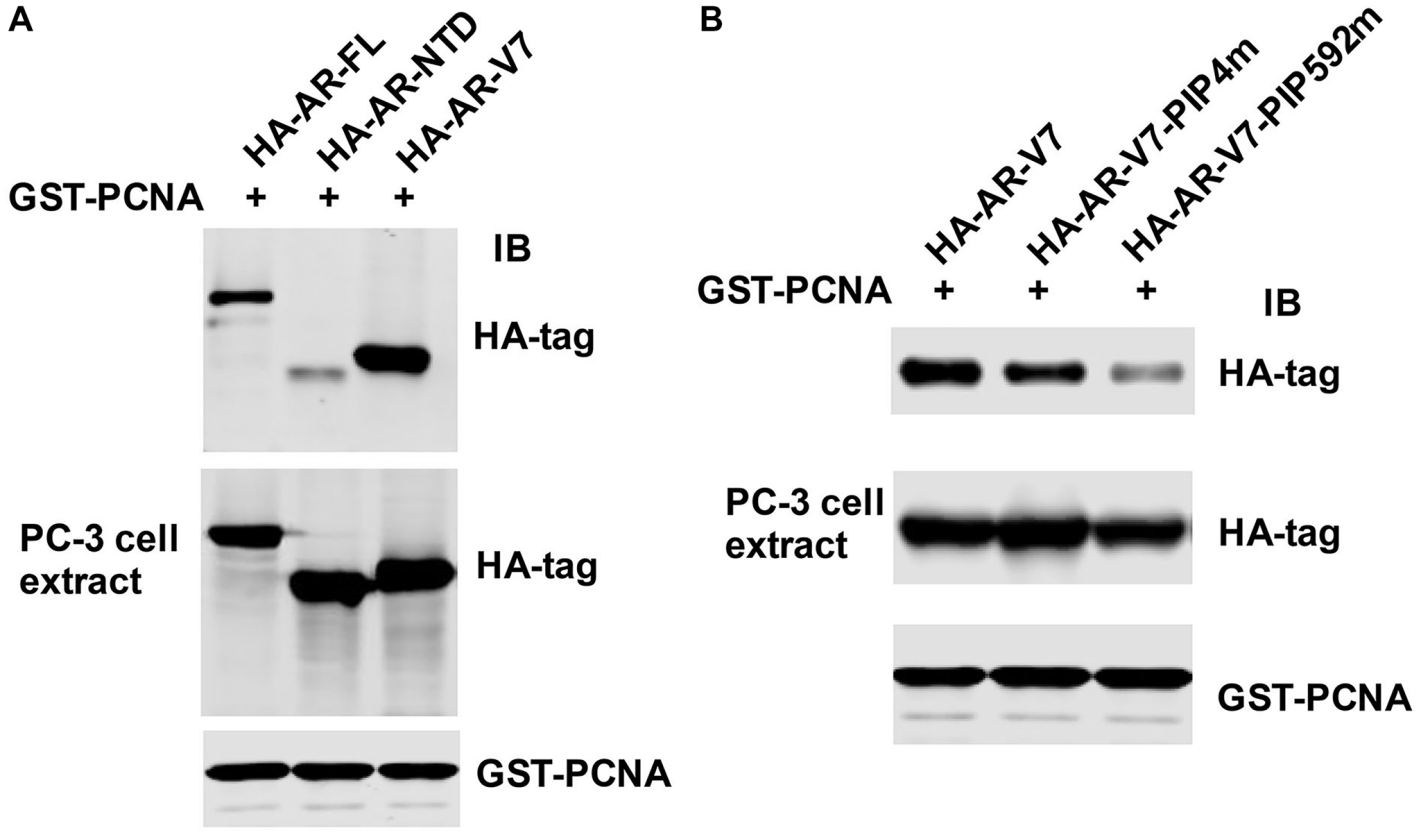
Identification of PIP-box592 at the DNA binding domain of AR. (**A**) The cell extracts from PC-3 transfected with HA-AR-FL, HA-AR-NTD, or HA-AR-V7 expression vectors and (**B**) the cell extracts from PC-3 transfected with HA-AR-V7, HA-AR-V7-PIP4m, or HA-AR-V7-PIP592m expression vectors were subjected to GST-PCNA pull-down assay (Upper panel). The transfected PC-3 cell extracts (25 μg) were served as loading control (Lower panel). HA-tag and PCNA antibodies were used for immunoblot analysis.

To further characterize the role of the PIP-box on PCNA/AR interaction, GST-PCNA pull-down assay was performed. Cell lysates from AR-negative PC-3 cells transfected with HA-tagged full-length AR (HA-AR-FL), AR N-terminal domain (HA-AR-NTD, AR1-539aa), or HA-AR-V7 (containing both the NTD and DBD, AR1-628 aa) were incubated with GST-PCNA, respectively (Figure 1). As shown in Figure 2A, HA-AR-NTD, containing AR PIP-box4 (^4^QLGLGRVY^11^), binds to PCNA, while HA-AR-V7 binds more strongly to PCNA in comparison with either HA-AR-NTD or HA-AR-FL. These data suggest that the DNA binding domain may contain an additional PIP-box.

We have identified a PIP-box4 (^4^QLGLGRVY^11^) at the N-terminus of AR [39]. Sequence analysis of the 65 amino acids in the DNA binding domain of AR revealed another potential consensus sequence of the PIP-box (^592^QKYLCASR^599^) at 592 amino acid position, named PIP-box592. We generated mutation constructs HA-AR-V7-PIP4m with deletion of eight amino acids PIP-box4 ^4^QLGLGRVY^11^ and HA-AR-V7-PIP592m with deletion of eight amino acids PIP-box592 ^592^QKYLCASR^599^, respectively, from HA-AR-V7 construct. To determine the role of PIP-box4 and PIP-box592 of AR in PCNA/AR interaction, we performed GST-PCNA pull-down assay using the cell lysates from AR negative PC-3 cells transfected with HA-AR-V7, HA-AR-V7-PIP4m, and HA-AR-V7-PIP592m, respectively. We found that deletion of PIP-box592 (^592^QKYLCASR^599^) significantly compromises the interaction of HA-AR-V7 with PCNA, while deletion of PIP-box4 moderately reduces the interaction (Figure 2B). This finding suggests that both PIP-box4 (^4^QLGLGRVY^11^) at the N-terminus and, more significantly, PIP-box592 (^592^QKYLCASR^599^) at the DNA binding domain contribute to the interaction of AR with PCNA.

### Dihydrotestosterone (DHT) only upregulates AR-FL binding to PCNA, but not AR-Vs

**Figure 3:**
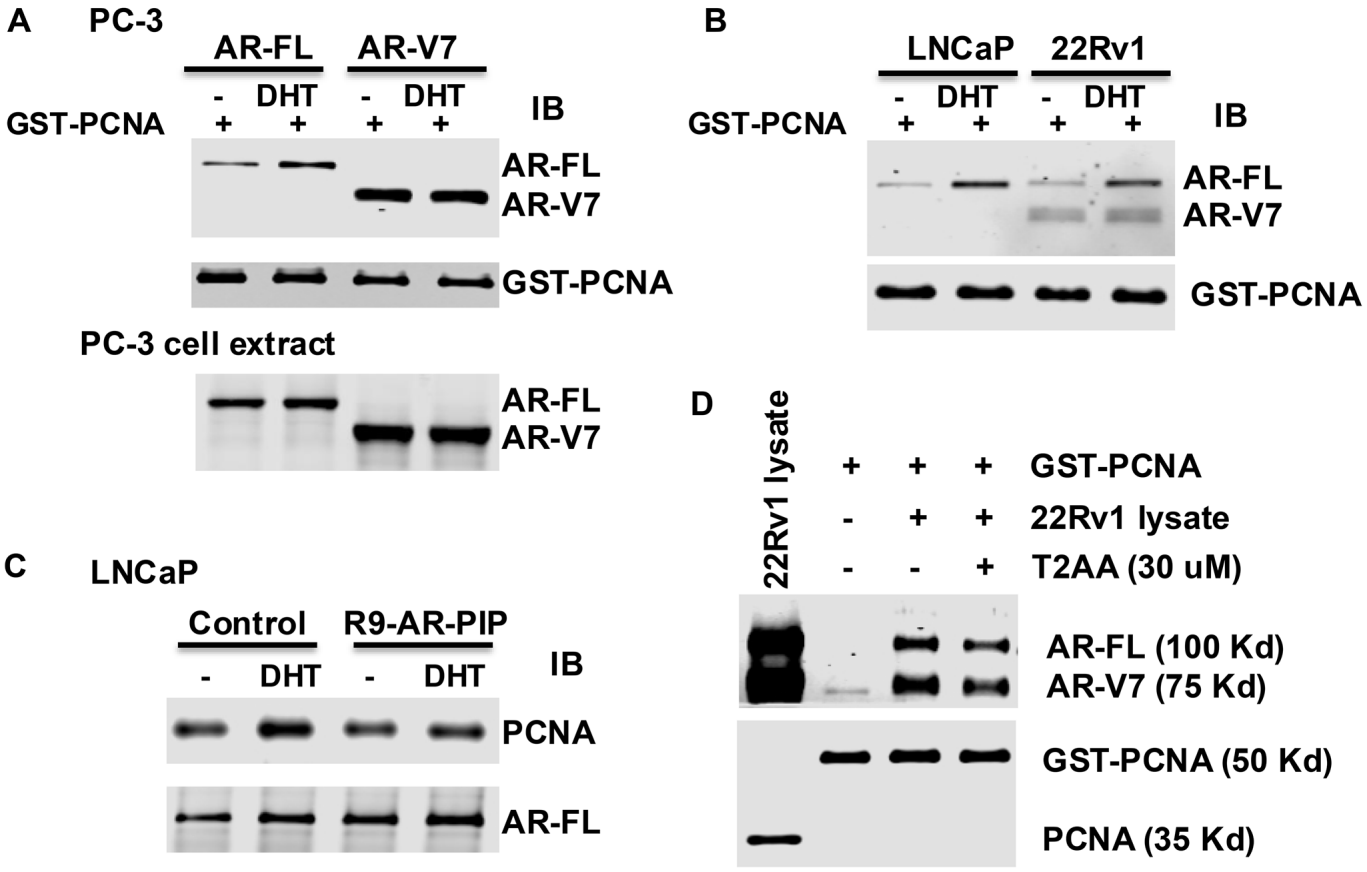
Dihydrotestosterone enhances the binding of AR-FL to PCNA. (**A**) PC-3 cells transfected with AR-FL or AR-V7 expression vector and (**B**) LNCaP and 22Rv1 cells, were treated without or with dihydrotestosterone (DHT) (10^−8^M) in stripped medium overnight. The cell extracts (1 mg) from these cells were subjected to GST-PCNA pull-down assay. AR(441) antibody was used for immunoblot analysis. Input of GST-PCNA for the pull-down assay was indicated. PC-3 cell extracts (25 μg) were served as loading control for GST pull-down (A, lower panel). (**C**, **D**) The cell extracts (1 mg) from LNCaP cells (C) without or with dihydrotestosterone and/or R9-AR-PIP (20 μM) treatment overnight were subjected to Co-Immunoprecipitation (Co-IP) using AR antibody. The cell extracts (1 mg) from 22Rv1 cells (D) were subjected to GST-PCNA pull-down assay in the absence and presence of 30 μM T2AA. PCNA and AR antibodies were used for immunoblot analysis.

Given that PIP-box4 and PIP-box592 are located at the N-terminus and DNA-binding domain of AR, respectively (Figures 1 and 2), we hypothesized that androgen regulates the interaction of AR-FL with PCNA. GST-PCNA pull-down assay was performed using the cell extracts from AR negative PC-3 cell transfected with AR-FL or AR-V7 expression vector without or with dihydrotestosterone. We found that dihydrotestosterone enhances the interaction of AR-FL, but not AR-V7, with PCNA (Figure 3A). To examine the endogenous AR-FL and AR-V7, cell extracts from LNCaP (AR-FL^+^) cells expressing only AR-FL and 22Rv1 (AR-FL^+^/AR-V7^+^) cells expressing both AR-FL and AR-V7 were used for GST-PCNA pull-down assay. As shown in Figure 3B, dihydrotestosterone also enhances the interaction of the endogenous AR-FL with PCNA in LNCaP and 22Rv1 cells but has no significant impact on AR-V7 binding to PCNA in 22Rv1 cells. Similarly, dihydrotestosterone enhances the interaction of AR-FL with PCNA in co-immunoprecipitation assay (Figure 3C). R9-AR-PIP significantly inhibits the interaction of AR-FL with PCNA in the presence of dihydrotestosterone (Figure 3C). The interaction of PCNA with AR-FL and AR-V7 is also attenuated by the small molecule PIP box inhibitor T2AA (Figure 3D).

### The role of the PIP-box on AR transcriptional activity

**Figure 4:**
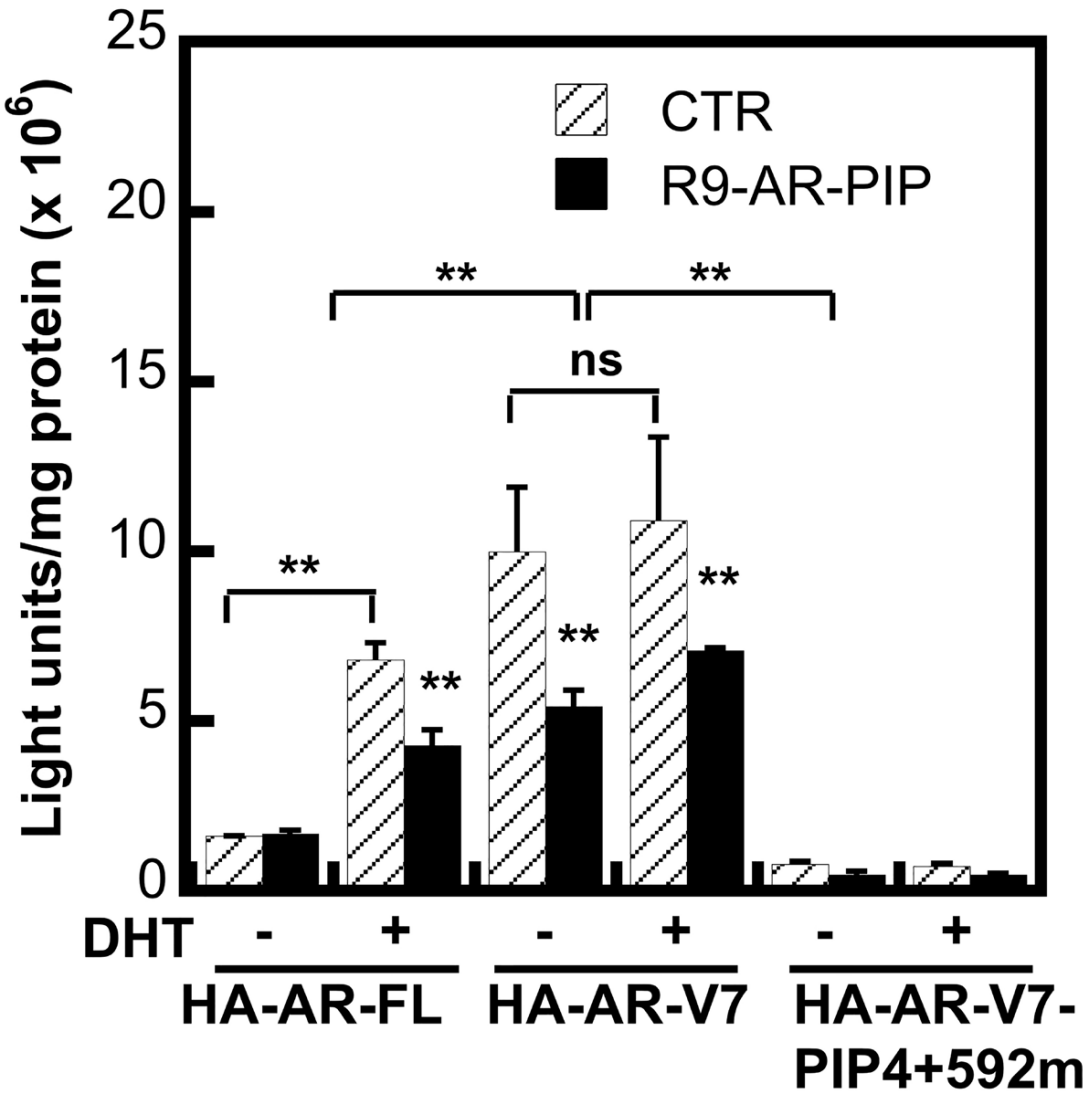
R9-AR-PIP inhibits transcriptional activity of AR-FL and AR-Vs. PC-3 cells transfected with PSA(4.3)-Luc reporter as well as expression vector HA-AR-FL, HA-AR-V7, or HA-AR-V7-PIP4+592m for 4 hours respectively, and then incubated without (control) or with R9-AR-PIP (20 μM) and/or dihydrotestosterone (DHT) (10^−8^M) for 24 hours, followed by luciferase assay.

We further investigate the role of the PIP-box on transcriptional activity of both AR-FL and AR-Vs using AR negative PC-3 cell co-transfecting prostate specific antigen (PSA) gene reporter PSA-Luc with AR-FL or AR-Vs expression vector in luciferase reporter assay. Data in Figure 4 shows that R9-AR-PIP significantly inhibits HA-AR-FL activity upon dihydrotestosterone stimulation, but has no effect on the inactive HA-AR-FL. R9-AR-PIP significantly inhibits HA-AR-V7 activity regardless of dihydrotestosterone stimulation. HA-AR-V7-PIP4+592m, a HA-AR-V7 with deletion of both PIP-box4 and PIP-box592, loses its constitutively active activity, and its basal activity is lower than that of the inactive HA-AR-FL (Figure 4). Consistently, R9-AR-PIP has no significant impact on the activity of HA-AR-V7-PIP4+592m. This data is consistent with PCNA/AR binding assay reported in Figure 3 and supports the notion in that the PIP-boxes in AR-V7 contributes to its constitutively active activity.

### Targeting PCNA/AR interaction compromises AR association to chromatin

**Figure 5:**
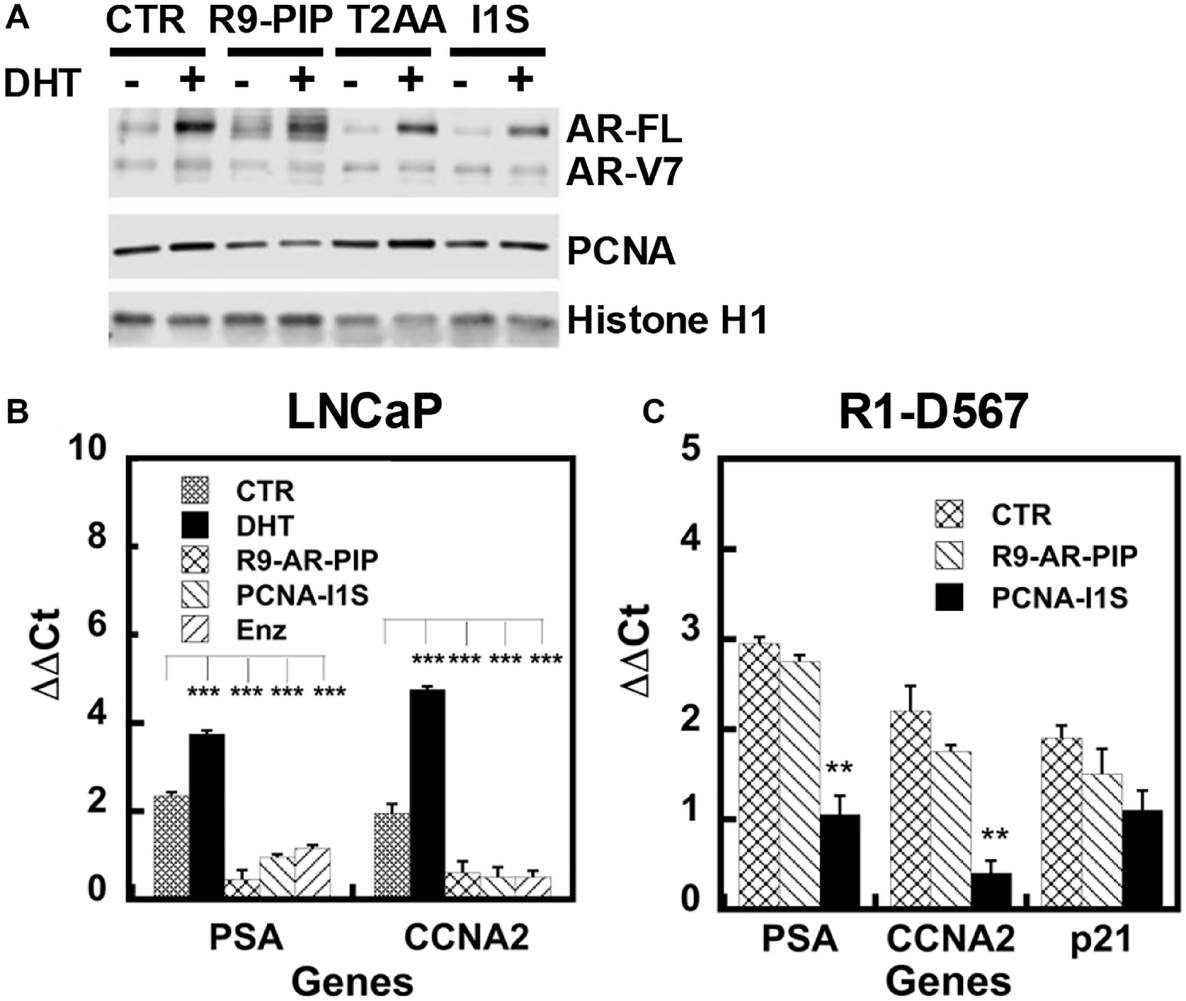
R9-AR-PIP and PCNA-I1S inhibit the chromatin association and the ARE occupancy by AR-FL and AR-Vs. (**A**) 22Rv1 cells in the stripped medium treated without or with dihydrotestosterone (DHT, 10^−8^M), R9-AR-PIP (R9-PIP, 20 μM), T2AA (5 μM), and/or PCNA-I1S (I1S, 1 μM) for 24 hours. Fifty mg chromatin-bound nuclear proteins were subjected to immunoblot analysis using AR(441), PCNA, and Histone H1 antibodies. LNCaP (**B**) or R1-D567 (**C**) cells in the stripped medium were treated without or with dihydrotestosterone (10^−8^M), R9-AR-PIP (20 μM), PCNA-I1S (1 μM), or Enzalutamide (Enz, 20 μM) overnight. These cells were subjected chromatin immunoprecipitation (ChIP) assay.

Since gene transcription occurs on chromatin, we assessed whether blocking PCNA/AR interaction compromises chromatin association of AR and PCNA by examining chromatin-bound nuclear proteins. Three PCNA inhibitors were used, including small peptideR9-AR-PIP, small molecule T2AA blocking the PIP-box, and PCNA-I1S interfering with PCNA re-localization to the nucleus and chromatin association for function. As shown in Figure 5A, dihydrotestosterone significantly enhances the association of AR-FL to chromatin, but moderately enhances the association by AR-V7, which may be due to autoregulation of AR expression by androgen [40]. R9-AR-PIP, T2AA, and PCNA-I1S significantly inhibit chromatin association for AR-FL and moderately for AR-V7 upon dihydrotestosterone stimulation. Consistent with what we reported before for PCNA-I1S [35, 36], R9-AR-PIP also significantly reduces the chromatin association of PCNA (Figure 5A).

### Targeting PCNA/AR interaction inhibits the ARE occupancy in AR target genes

We determined the ARE occupancy by AR in AR target genes *p21(WAF), PSA*, and *CCNA2* using the chromatin immunoprecipitation assay. In LNCaP cells, dihydrotestosterone enhances and R9-AR-PIP, PCNA-I1S, and enzalutamide inhibit the ARE occupancy by AR-FL in canonical AR target gene PSA as well as *CCNA2* gene (Figure 5B). In R1-D567 cells expressing only ARv567es, PCNA-I1S significantly inhibits the ARE occupancy in *PSA* and *CCNA2* genes (Figure 5C). These data indicate that blocking PCNA/AR-FL and PCNA/AR-Vs interaction compromise the ARE occupancy by AR.

### Targeting PCNA/AR interaction inhibits co-localization of AR with PCNA

**Figure 6:**
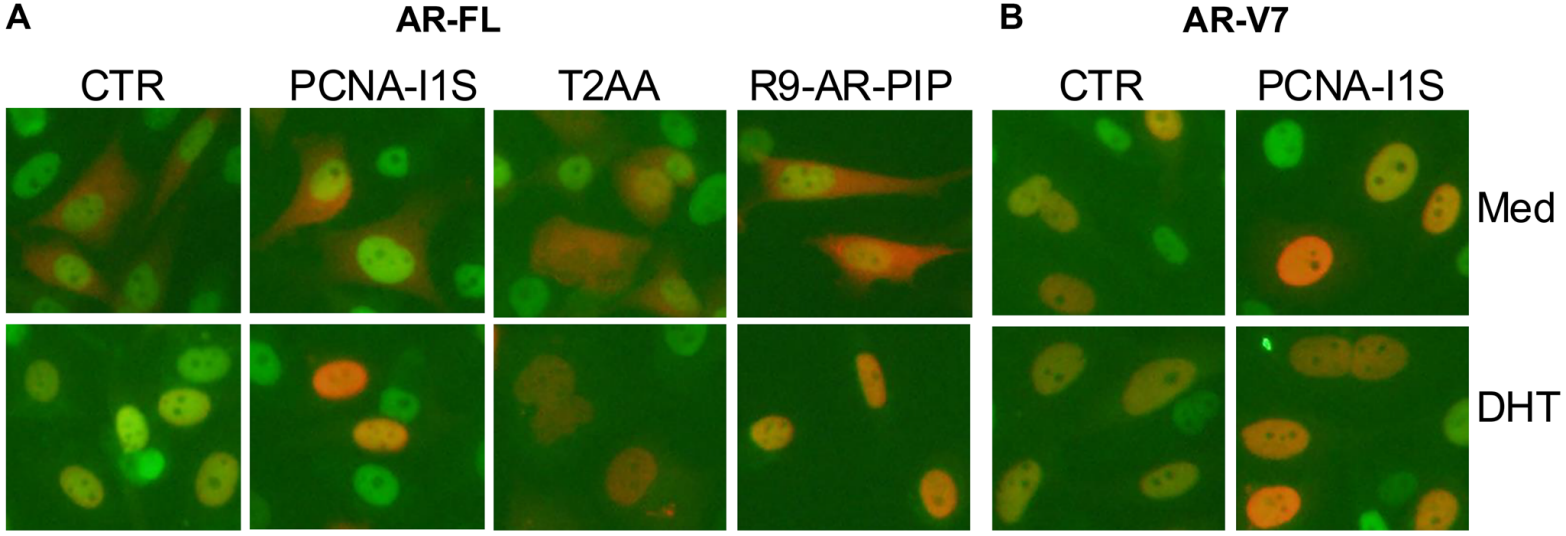
Colocalization of AR-FL and AR-V7 with PCNA. PC-3 cells in stripped medium were transfected with expression vector AR-FL (**A**) or AR-V7 (**B**) for 18 hours, incubated either in medium (CTR) or treated with PCNA-I1S (1 μM), T2AA (20 μM), or R9-AR-PIP (20 μM) for 1 hour, then followed by incubation in medium (Med) or stimulation with dihydrotestosterone (DHT) (10 nM) for 4 hours. Subsequently, the cells were stained with fluorophore-labeled anti-AR reacting with both AR-FL and AR-V7 (Alexa Fluor594, red color) and ani-PCNA (Alexa Fluor488, green color) antibodies.

We investigated the co-localization of AR-FL and AR-V7 with PCNA in AR negative PC-3 cells transfected with expression vectors AR-FL and AR-V7, followed by immunofluorescent staining. As shown in Figure 6A, AR-FL (red color) in the transfected cells is detected mainly in the cytoplasm and PCNA (green color) is mainly located in the nucleus. Upon dihydrotestosterone stimulation, AR-FL is re-localized to the nucleus and co-localized with PCNA demonstrated as yellow color due to AR-FL and PCNA interaction. Treatment with PCNA inhibitors PCNA-I1S, T2AA, or R9-AR-PIP alone does not affect AR-FL or PCNA localization but attenuates co-localization of AR-FL and PCNA as indicated by a reduced yellow color upon dihydrotestosterone stimulation (Figure 6A). On the other hand, AR-V7 is detected almost exclusively in the nucleus regardless of presence or absence of dihydrotestosterone, and its co-localization with PCNA is also attenuated by PCNA-I1S (Figure 6B), as well as by T2AA, or R9-AR-PIP (data not shown). Similar results are observed in LNCaP cells expressing AR-FL (data not shown). Therefore, R9-AR-PIP and PCNA-I1S attenuate co-localization of AR-FL with PCNA upon dihydrotestosterone stimulation as well as AR-V7 with PCNA regardless of presence or absence of dihydrotestosterone.

### Targeting PCNA/AR interaction induces cytotoxicity in CRPC cells

**Figure 7:**
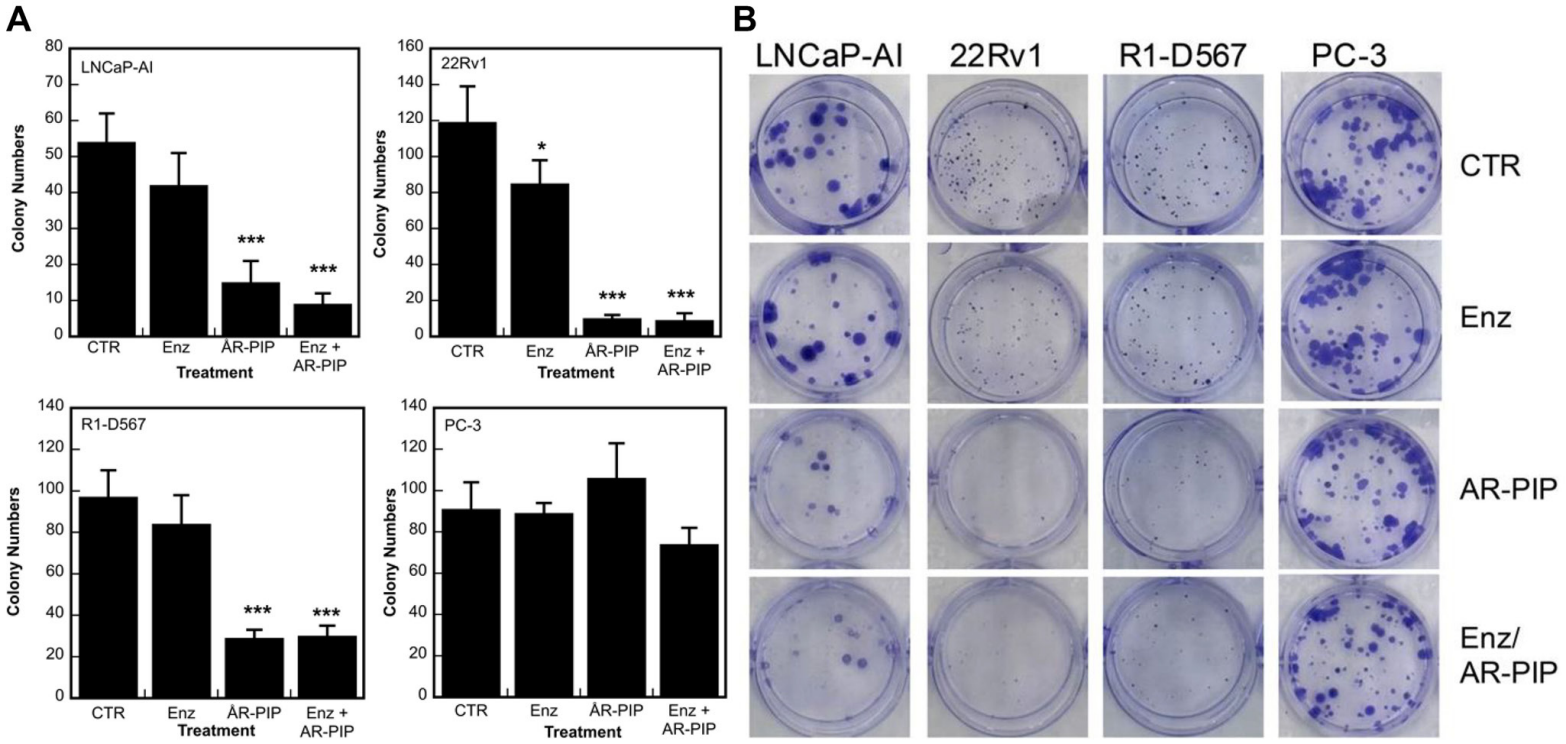
The differential cytotoxic effects of PCNA inhibitors in prostate cancer cells varying with AR expression status. (**A**) Cells were plated into 12-well plates at 300–500 cells/well. After an overnight incubation, the cells were treated with enzalutamide (Enz, 20 μM), R9-AR-PIP (R9-PIP, 20 μM), or Enz plus R9-AR-PIP. Twenty-four hours later, the cells were cultured in fresh medium for up to 2 weeks. The colonies formed by these cells were stained with crystal violet and counted using ImageJ with Colony Counter plug-in. (**B**) Representative images of the assay. ns, not significant; ^*^ *p* < 0.05; ^***^ *p* < 0.001.

The cytotoxicity was assessed in the classic colony formation (clonogenic cell survival) assay using four lines of CRPC cells with differential expression of AR, including LNCaP-AI (AR-FL^+^), 22Rv1(AR-FL^+^/AR-V7^+^), R1-D567 (Arv567es), and PC-3(AR^−^) cells, as described in our previous study [38]. As shown in Figure 7A, treatment with R9-AR-PIP attenuates colony formation in three AR positive LNCaP-AI, 22Rv1, and R1-D567 cells, but not significantly in AR negative PC-3 cells. Enzalutamide induces cytotoxicity only in 22Rv1 cells that express both AR-FL and AR-V7 (Figure 7A). The treatment with enzalutamide and R9-AR-PIP produces moderate additive effects in induction of cytotoxicity in LNCaP-AI cells (Figure 7A). Representative images of the clonogenic assay in the four lines of CRPC cells are shown in Figure 7B.

### Dihydrotestosterone stimulates cyclin A2 expression

**Figure 8:**
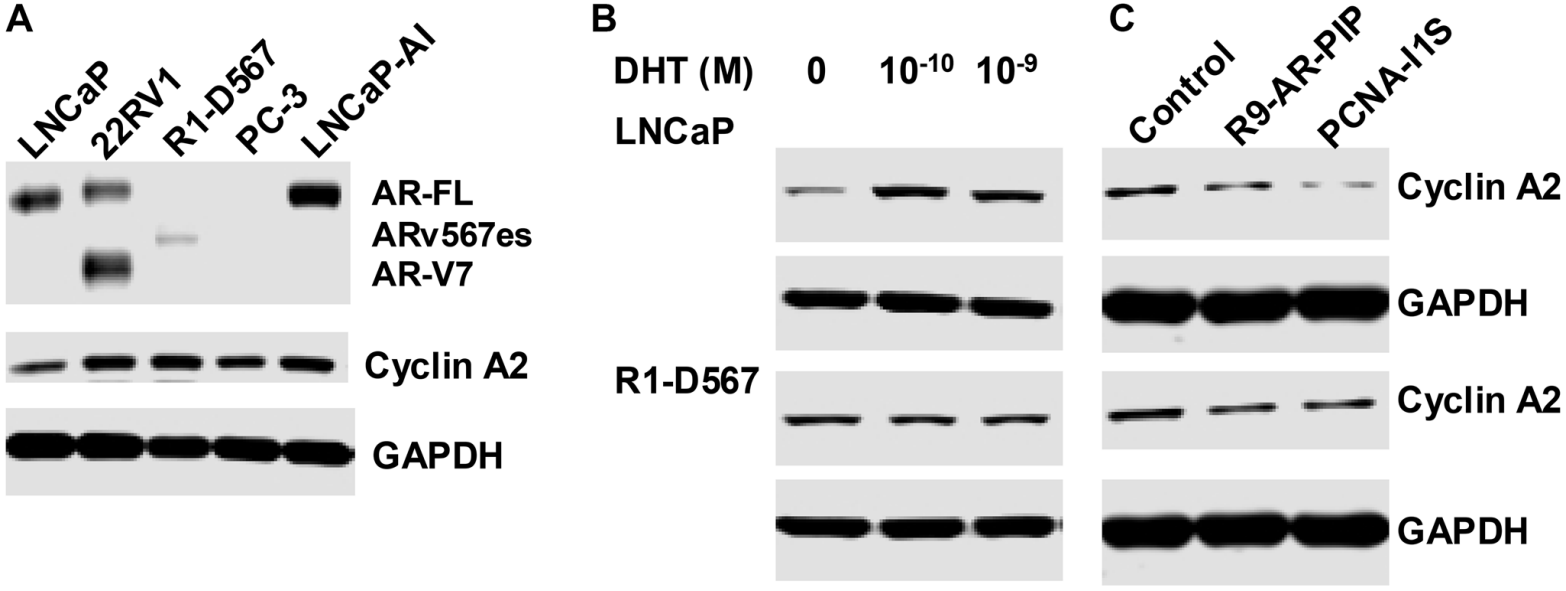
Dihydrotestosterone regulates cyclin A2 expression in AR-FL expressing cells. (**A**) Cell extracts from androgen dependent LNCaP (AR-FL^+^) cells and 4 lines of CRPC cells LNCaP-AI (AR-FL^+^), 22Rv1(AR-FL^+^/AR-V7^+^), R1-D567 (ARv567es), and PC-3(AR^−^) were prepared. LNCaP or R1-D567 cells were cultured in stripped medium for 1 day and then treated without or with dihydrotestosterone (DHT) (**B**), as well as R9-AR-PIP (20 μM) or PCNA-I1S (1 μM) (**C**) for 24 hours. The cell extracts from these cells were subjected to immunoblot analysis using AR, cyclin A, and GAPDH antibodies.

Cyclin A2 was reported as an AR-Vs specific target gene in prostate cancer cells [41–43]. Given that dihydrotestosterone enhances the ARE occupancy by AR-FL in *CCNA2* gene (Figure 5C), we evaluated whether dihydrotestosterone regulates cyclin A2 expression. Cyclin A2 protein is elevated in all CRPC cells, including LNCaP-AI (AR-FL^+^), 22Rv1(AR-FL^+^/AR-V7^+^), R1-D567 (ARv567es), and PC-3(AR^−^) cells, as compared with androgen dependent LNCaP (AR-FL^+^) cells (Figure 8A). Dihydrotestosterone stimulates cyclin A2 expression in LNCaP, but not in R1-D567 cells (Figure 8B). R9-AR-PIP and PCNA-I1S attenuate cyclin A2 expression in both LNCaP and R1-D567 cells (Figure 8C). Therefore, targeting PCNA/AR interaction compromises both AR-FL and AR-Vs signaling-mediated upregulation of cyclin A2 expression.

## DISCUSSION

We identified a novel PIP-box592 (^592^QKYLCASR^599^) at the DNA binding domain of AR, in addition to previously discovered PIP-box4 at the N-terminus of AR [39]. The PIP-boxes contribute to the transcriptional activity of AR-FL upon dihydrotestosterone stimulation. Upon dihydrotestosterone binding, AR-FL exposes the PIP-box592 at the DNA binding domain, leading to an enhanced interaction with PCNA and its activity (Figure 3) [44]. This finding is the first to demonstrate that steroid hormone regulates the interaction of AR-FL with PCNA. Furthermore, AR-V7 with deletion of both PIP-box4 and PIP-box592 loses its constitutively active activity. Due to lacking the ligand binding domain and presence of nucleus localization signal (Figure 1), AR-V7 with exposed PIP-box592 can be localized to the nucleus and freely interact with PCNA, which may contribute to its constitutively active function. This finding reveals a novel underlying mechanism of constitutively active AR-Vs and an aberrant elevated AR signaling in CRPC due to overexpression of AR-V7 and PCNA. Importantly, both AR-FL and AR-Vs activity can be attenuated by targeting AR/PCNA interaction (Figures 4 and 5).

Androgen regulates cell cycle genes, such as canonical AR target gene cdc6 [45]. AR-Vs bind to the ARE and upregulate the canonical AR target genes related to metabolism, secretion, and differentiation, such as *PSA* and *FKBP5*, as well as AR-Vs-specific or target genes, such as *UBE2C* and *CCNA2* associated with cell cycle progression [41–43, 46]. However, our understanding of differential AR-Vs-driven gene transcription relative to AR-FL is limited. FOXA1 was suggested to physically interact with AR as a cofactor and recruit AR-FL or AR-Vs to low-affinity ARE half site adjacent to Fox A1 motifs [41]. On the other hand, AR-Vs are dependent on FOXA1 for sustaining a pro-proliferative gene signature, including *CCNA2 gene*, encoding cyclin A2. Together with CDK1 or CDK2, cyclin A2 promotes both G1/S and G2/M transitions in somatic cells [47]. Several data-mining analyses revealed that cyclin A2 is overexpressed in prostate cancer and its overexpression is associated with poor prognosis and short overall cancer survival [48–51]. Cyclin A2 was suggested to be a biomarker and therapeutic target for advanced prostate cancer [49, 51]. Indeed, knockdown expression of cyclin A2 using siRNA or inhibition of cyclin A2 activity using small molecule were shown to inhibit cell growth, invasion, and migration in several lines of prostate cancer cells [49, 51, 52]. Although it was reported that *CCNA2* is an AR-Vs specific or target gene in prostate cancer cells [41–43], our finding reveals that dihydrotestosterone increases the ARE occupancy by AR-FL in *CCNA2* gene and cyclin A2 expression in AR-FL positive cells. Furthermore, R9-AR-PIP and PCNA-I1S reduce cyclin A2 expression in prostate cancer cells expressing both AR-FL and AR-Vs. These data suggest that *CCNA2* gene is a canonical AR target gene. Cyclin A2 is overexpressed in all CRPC cell lines examined, regardless of their AR expression statuses, as compared with androgen dependent cells, suggesting that it contributes to the development of CRPC.

AR-V7 expression is very low in the normal prostate gland, increased in tumors, and further elevated in CRPC. Nuclear AR-V7 emerges with CRPC in 75% of cases relative to those in less than 1% prior to therapy and is further increased upon treatment with abiraterone or enzalutamide [53]. Our recent study shows that targeting PCNA/AR interaction with enzalutamide, PCNA-I1S, R9-AR-PIP, and/or T2AA induces cytotoxicity in both androgen-dependent prostate cancer cells and CRPC cells [54]. Apoptosis, which is observed in AR expressing cells treated with AR-targeting small peptide [55], AR inhibitor enzalutamide [56], or PCNA-targeting small molecule inhibitor [38], could be a main underlying mechanism causing the cytotoxicity observed in our study. Data presented in this study show that targeting PCNA/AR interaction attenuates both AR-FL- and AR-Vs-mediated signaling and cyclin A2 expression and induces cytotoxicity in CRPC cells. This finding implicates that targeting PCNA/AR interaction could be an innovative strategy for therapy against CRPC. Further studies in animal models are needed to validate the effects of targeting PCNA/AR interaction on tumor growth, and cyclin A2 and AR expression in tumor lesions.

## MATERIALS AND METHODS

### Reagents

Dihydrotestosterone (DHT) and T2AA were purchased from Sigma-Aldrich (St Louis, MO, USA). Antibodies against mouse PCNA(PC10), rabbit PCNA(D3H8P), HA-tag, AR(D6F11), Histone H1, and cyclin A2(E6D1J) were purchased from Cell Signaling Technology, Inc (Danvers, MA, USA). Antibodies against AR (441) and GAPDH (sc-47724) were obtained from Santa Cruz Biotechnology, Inc (Santa Cruz, CA, USA). The GST-PCNA expression plasmid was generously provided by Dr. Shaochun Wang (University of Cincinnati, Cincinnati, OH, USA). PCNA-I1 and PCNA-I1S [35–37] were purchased from ChemBridge Corporation (San Diego, CA, USA). R9-AR-PIP was synthesized by the custom peptide service at Biomatik Corporation (Ontario, N2C 1N6, Canada). Plasmids AR-FL, AR-V7 (1-660 amino acid containing the NTD and DBD of AR, named as AR-V7 with additional 32 amino acid), HA-AR-FL, HA-AR-NTD (1-539 amino acid), and HA-AR-V7 (1-628 amino acid) were obtained from Addgene (Watertown, MA, USA) (Figure 1). Primers for Chromatin Immunoprecipitation (ChIP) PCR were synthesized at GeneScript (Piscataway, NJ, USA). The primer pair for p21 gene core promoter (forward: aagtgccctcctgcagcacg; reverse CAGCTGCTCACACCT CAGCTG) includes the ARE sequence as described in our previous publications [57, 58]. The primer pairs for CCNA2 (forward: TTAGTGAGCTGTCCAGTGACTCAAT; reverse: CCCATGTATTAAAGTAGCTTCTGT AAACA) and PSA (forward: CCTAGATGAAGTCT CCATTGAGCTACA; reverse: GGGAGGGA GAGCTAGCACTTG) genes are published by others [42].

### Generation of PIP-box deletion constructs

PIP-Box deletion constructs HA-AR-V7-PIP4m, HA-AR-V7-592m, and HA-AR-V7-PIP4+592m with deletion of PIP-box4 (^4^QLGLGRVY^11^), PIP-box592 (^592^QKYLCASR^599^), and both PIP-boxes, respectively, were generated from HA-AR-V7 using the Site-Directed Mutagenesis kit (ThermoFisher Scientific, Waltham, MA, USA).

### Cells and cell culture

The prostate cancer cell lines, LNCaP (CRL-1740), PC-3 (CRL-1435), and 22Rv1 (CRL-2505), were obtained from ATCC (Manassas, Virginia, USA). CWR-R1-D567 (R1-D567) cells [59] were obtained from Kerafast (Shirley, MA, USA). The androgen-independent LNCaP-AI cells we established were derived from LNCaP cells by long-term culture in stripped medium containing RPMI-1640 medium supplemented with 10% charcoal-dextran treated FBS (sFBS) [10]. The cells were authenticated genetically with PCR identifying the short tandem repeat (STR) and cell-specific profiling against the ATCC database at the University of Arizona Genetics Core. LNCaP, PC-3, R1-D567, and 22Rv1 cells were cultured in RPMI-1640 medium supplemented with 10% fetal bovine serum (FBS) at 37°C in 5% CO_2_.

### Immunoblot analysis

Immunoblot analysis was performed as previously described [60]. Briefly, aliquots of samples with the same amount of protein, determined using the DC Protein assay kit (Bio-Rad, Hercules, California, USA), were mixed with loading buffer (62.5 mM Tris-HCl, pH 6.8, 2.3% SDS, 100 mM dithiothreitol and 0.005% bromophenol blue), boiled, fractionated in SDS-PAGE, and transferred onto 0.45-μm nitrocellulose membranes (Bio-Rad Laboratories, Inc.). The membranes were blocked with 2% fat-free milk in PBS for one hour and probed with primary antibody in hybridization buffer (PBS containing 0.01% Tween-20 and 1% fat-free milk) at 4°C on a rocker platform overnight. The membranes were then washed four times in PBS and incubated with IRDye 680LT secondary antibodies (LI-COR Biosciences. Lincoln, NE, USA) with 1:1000 dilution in hybridization buffer on a rocker platform for one hour. After washing four times in PBS, the membranes were visualized using Odyssey imaging system (Li-COR).

### Co-immunoprecipitation assay

Co-immunoprecipitation was performed in a modified radio-immunoprecipitation assay (RIPA) buffer (PBS, 0.1% NP-40, 0.1% sodium deoxycholate, 50 mM NaCl, 1 mM EDTA, 1 mM dithiothreitol, 1 mM phenylmethylsulfonyl fluoride (PMSF), and 1X protease inhibitor cocktail). The antibody was incubated with cell extract (1 mg) at 4°C for 2 hours, followed by addition of 50 μl of Protein G plus/protein A agarose beads (ThermoFisher Scientific) and incubation at 4°C overnight. The beads were washed four times with the modified RIPA lysis buffer, harvested by spinning at 3500 rpm for 5 minutes, and boiled in SDS-PAGE loading buffer. The protein samples in the supernatant were subjected to SDS-PAGE and immunoblot analysis.

### GST-PCNA pull-down assay

GST-PCNA protein (5–10 μg), prepared in our lab as described previously [39], was incubated with 1 mg of cell extract in the modified RIPA buffer at 4°C on a rocker platform overnight. The beads were washed with the modified RIPA buffer for four times and boiled in SDS-PAGE loading buffer. The protein samples were subjected to SDS-PAGE and immunoblot analysis.

### Chromatin association assay

Cells in the stripped medium were treated without or with DHT, R9-AR-PIP, T2AA, and/or PCNA-I1S for 24 hours. The chromatin-bound nuclear proteins were isolated using the Subcellular Protein Fractionation Kit for Cultured Cells (ThermoFisher Scientific). 50 μg from each sample was subjected to immunnoblot analysis.

### Chromatin immunoprecipitation (ChIP) assay

Cells from 100-mm tissue culture plates were pelleted and subjected to DNA isolation using the Pierce Magnetic ChIP Kit (ThermoFisher Scientific), AR(D6F11) antibody, and/or IgG. DNA (3 μl) from each sample was subjected to quantitative PCR (qPCR) using the Fast SYBR-Green Master Mix and 7300 Real-Time PCR system (Applied Biosystems, Thermo Fisher Scientific). The qPCR condition is as following: denature at 95°C for 3 seconds and anneal/extend at 60°C for 30 seconds with total of 40 cycles. Input and recovered products after ChIP were normalized to the respective negative control (IgG) using the formula ΔCt = Ct_target product or input_ – Ct_IgG_. Comparative ΔΔCt values (fold enrichment) = ΔCt_target product_/ΔCt_input_, which was as described before [61].

### Reporter assay

PC-3 cells (100,000/well) were seeded in 24-well tissue culture plates. The next day, the cells were transfected with reporter plasmids (400 ng) and AR expression vector (400 ng) using Lipofectamine 3000^®^ reagent (ThermoFisher Scientific) for 4 hours. The cells were then treated with PCNA-I1S, R9-AR-PIP, and/or DHT for 24 hours. Glo lysis buffer (100 μl, Promega) was added to each well and incubated with cells for 5 minutes. Subsequently, 50 μl of the cell lysis was mixed with 50 μl Steady-Glo luciferase substrate (Promega, Madison, WI, USA) and was subjected to Luciferase assay using Tecan plate reader for luminescent assay. The luciferase activity was defined as relative light units/mg protein.

### Immunofluorescent staining

The immunofluorescent staining of cells was performed as described in our previous publication [38] with minor modifications. After incubation with primary antibodies, the cells were stained with fluorophore-labeled anti-AR (Alexa Fluor594) and ani-PCNA (Alexa Fluor488) antibodies (Cell Signaling Technology) and mounted for analysis under an Olympus fluorescent microscopy. Images were captured with an Olympus DP80 camera using the cellSens imaging software (v1.18).

### Colony formation assay

Colony formation was assessed as described in our previous publication with minor modifications [38]. Briefly, cells were in 12-well plates were incubated in medium or treated for 24 hours with enzalutamide and/or R9-AR-PIP, followed by culturing in fresh medium for up to 2 weeks. The colonies formed by the cells were stained with crystal violet and counted using ImageJ with Colony Counter plug-in.

### Statistical analysis

All experiments were repeated two to three times. Data from each assay are expressed as the mean ± SD. Statistically significant differences between two groups were determined using the Student’s *t*-test from a representative experiment. All statistical analyses were carried out using Prism 9 software (GraphPad, Inc.). *P* < 0.05 was considered to indicate a statistically significant difference.

## Abbreviations

AR: androgen receptor
AR-FL: full-length AR
AR-Vs: constitutively active AR splicing variants
CRPC: castration resistant prostate cancer
DBD: DNA binding domain
LBD: ligand binding domain
PCNA: proliferating cell nuclear antigen
PIP-box: PCNA interacting protein box
PCNA: inhibitors
PSA: prostate specific antigen

## References

[1] Choi E , Buie J , Camacho J , Sharma P , de Riese WTW . Evolution of Androgen Deprivation Therapy (ADT) and Its New Emerging Modalities in Prostate Cancer: An Update for Practicing Urologists, Clinicians and Medical Providers. Res Rep Urol. 2022; 14:87–108. 10.2147/RRU.S303215. 35386270 PMC8977476

[2] Litwin MS , Tan HJ . The Diagnosis and Treatment of Prostate Cancer: A Review. JAMA. 2017; 317:2532–42. 10.1001/jama.2017.7248. 28655021

[3] Gourdin T . Highlighting recent progress in the treatment of men with advanced prostate cancer. Curr Opin Oncol. 2024; 36:174–79. 10.1097/CCO.0000000000001035. 38573207

[4] Daniels VA , Luo J , Paller CJ , Kanayama M . Therapeutic Approaches to Targeting Androgen Receptor Splice Variants. Cells. 2024; 13:104. 10.3390/cells13010104. 38201308 PMC10778271

[5] Krause WC , Shafi AA , Nakka M , Weigel NL . Androgen receptor and its splice variant, AR-V7, differentially regulate FOXA1 sensitive genes in LNCaP prostate cancer cells. Int J Biochem Cell Biol. 2014; 54:49–59. 10.1016/j.biocel.2014.06.013. 25008967 PMC4160387

[6] Ciccarese C , Santoni M , Brunelli M , Buti S , Modena A , Nabissi M , Artibani W , Martignoni G , Montironi R , Tortora G , Massari F . AR-V7 and prostate cancer: The watershed for treatment selection? Cancer Treat Rev. 2016; 43:27–35. 10.1016/j.ctrv.2015.12.003. 26827690

[7] Paschalis A , Sharp A , Welti JC , Neeb A , Raj GV , Luo J , Plymate SR , de Bono JS . Alternative splicing in prostate cancer. Nat Rev Clin Oncol. 2018; 15:663–75. 10.1038/s41571-018-0085-0. 30135575

[8] Dehm SM , Tindall DJ . Alternatively spliced androgen receptor variants. Endocr Relat Cancer. 2011; 18:R183–96. 10.1530/ERC-11-0141. 21778211 PMC3235645

[9] Wadosky KM , Koochekpour S . Molecular mechanisms underlying resistance to androgen deprivation therapy in prostate cancer. Oncotarget. 2016; 7:64447–70. 10.18632/oncotarget.10901. 27487144 PMC5325456

[10] Lu S , Tsai SY , Tsai MJ . Molecular mechanisms of androgen-independent growth of human prostate cancer LNCaP-AI cells. Endocrinology. 1999; 140:5054–59. 10.1210/endo.140.11.7086. 10537131

[11] Yin Y , Li R , Xu K , Ding S , Li J , Baek G , Ramanand SG , Ding S , Liu Z , Gao Y , Kanchwala MS , Li X , Hutchinson R , et al. Androgen Receptor Variants Mediate DNA Repair after Prostate Cancer Irradiation. Cancer Res. 2017; 77:4745–54. 10.1158/0008-5472.CAN-17-0164. 28754673 PMC5600864

[12] Le TK , Duong QH , Baylot V , Fargette C , Baboudjian M , Colleaux L , Taïeb D , Rocchi P . Castration-Resistant Prostate Cancer: From Uncovered Resistance Mechanisms to Current Treatments. Cancers (Basel). 2023; 15:5047. 10.3390/cancers15205047. 37894414 PMC10605314

[13] Deshmukh RR , Schmitt SM , Hwang C , Dou QP . Chemotherapeutic inhibitors in the treatment of prostate cancer. Expert Opin Pharmacother. 2014; 15:11–22. 10.1517/14656566.2014.852184. 24156780

[14] Gebrael G , Fortuna GG , Sayegh N , Swami U , Agarwal N . Advances in the treatment of metastatic prostate cancer. Trends Cancer. 2023; 9:840–54. 10.1016/j.trecan.2023.06.009. 37442702

[15] McKay RR , Morgans AK , Shore ND , Dunshee C , Devgan G , Agarwal N . First-line combination treatment with PARP and androgen receptor-signaling inhibitors in HRR-deficient mCRPC: Applying clinical study findings to clinical practice in the United States. Cancer Treat Rev. 2024; 126:102726. 10.1016/j.ctrv.2024.102726. 38613872

[16] Naryzhny SN , Desouza LV , Siu KW , Lee H . Characterization of the human proliferating cell nuclear antigen physico-chemical properties: aspects of double trimer stability. Biochem Cell Biol. 2006; 84:669–76. 10.1139/o06-037. 17167529

[17] Naryzhny SN . Proliferating cell nuclear antigen: a proteomics view. Cell Mol Life Sci. 2008; 65:3789–808. 10.1007/s00018-008-8305-x. 18726183 PMC11131649

[18] Krishna TS , Kong XP , Gary S , Burgers PM , Kuriyan J . Crystal structure of the eukaryotic DNA polymerase processivity factor PCNA. Cell. 1994; 79:1233–43. 10.1016/0092-8674(94)90014-0. 8001157

[19] Gulbis JM , Kelman Z , Hurwitz J , O’Donnell M , Kuriyan J . Structure of the C-terminal region of p21(WAF1/CIP1) complexed with human PCNA. Cell. 1996; 87:297–306. 10.1016/s0092-8674(00)81347-1. 8861913

[20] Stoimenov I , Helleday T . PCNA on the crossroad of cancer. Biochem Soc Trans. 2009; 37:605–13. 10.1042/BST0370605. 19442257

[21] Witko-Sarsat V , Mocek J , Bouayad D , Tamassia N , Ribeil JA , Candalh C , Davezac N , Reuter N , Mouthon L , Hermine O , Pederzoli-Ribeil M , Cassatella MA . Proliferating cell nuclear antigen acts as a cytoplasmic platform controlling human neutrophil survival. J Exp Med. 2010; 207:2631–45. 10.1084/jem.20092241. 20975039 PMC2989777

[22] Naryzhny SN , Lee H . Proliferating cell nuclear antigen in the cytoplasm interacts with components of glycolysis and cancer. FEBS Lett. 2010; 584:4292–98. 10.1016/j.febslet.2010.09.021. 20849852

[23] Ohayon D , De Chiara A , Chapuis N , Candalh C , Mocek J , Ribeil JA , Haddaoui L , Ifrah N , Hermine O , Bouillaud F , Frachet P , Bouscary D , Witko-Sarsat V . Cytoplasmic proliferating cell nuclear antigen connects glycolysis and cell survival in acute myeloid leukemia. Sci Rep. 2016; 6:35561. 10.1038/srep35561. 27759041 PMC5069676

[24] Gilljam KM , Feyzi E , Aas PA , Sousa MM , Müller R , Vågbø CB , Catterall TC , Liabakk NB , Slupphaug G , Drabløs F , Krokan HE , Otterlei M . Identification of a novel, widespread, and functionally important PCNA-binding motif. J Cell Biol. 2009; 186:645–54. 10.1083/jcb.200903138. 19736315 PMC2742182

[25] Mailand N , Gibbs-Seymour I , Bekker-Jensen S . Regulation of PCNA-protein interactions for genome stability. Nat Rev Mol Cell Biol. 2013; 14:269–82. 10.1038/nrm3562. 23594953

[26] Olaisen C , Müller R , Nedal A , Otterlei M . PCNA-interacting peptides reduce Akt phosphorylation and TLR-mediated cytokine secretion suggesting a role of PCNA in cellular signaling. Cell Signal. 2015; 27:1478–87. 10.1016/j.cellsig.2015.03.009. 25797046

[27] Lu S , Lee J , Revelo M , Wang X , Lu S , Dong Z . Smad3 is overexpressed in advanced human prostate cancer and necessary for progressive growth of prostate cancer cells in nude mice. Clin Cancer Res. 2007; 13:5692–702. 10.1158/1078-0432.CCR-07-1078. 17908958

[28] Spires SE , Banks ER , Davey DD , Jennings CD , Wood DP Jr , Cibull ML . Proliferating cell nuclear antigen in prostatic adenocarcinoma: correlation with established prognostic indicators. Urology. 1994; 43:660–66. 10.1016/0090-4295(94)90181-3. 7909398

[29] Kallakury BV , Sheehan CE , Rhee SJ , Fisher HA , Kaufman RP Jr , Rifkin MD , Ross JS . The prognostic significance of proliferation-associated nucleolar protein p120 expression in prostate adenocarcinoma: a comparison with cyclins A and B1, Ki-67, proliferating cell nuclear antigen, and p34cdc2. Cancer. 1999; 85:1569–76. 10.1002/(sici)1097-0142(19990401)85:7<1569::aid-cncr19>3.0.co;2-m. 10193948

[30] Harper ME , Glynne-Jones E , Goddard L , Wilson DW , Matenhelia SS , Conn IG , Peeling WB , Griffiths K . Relationship of proliferating cell nuclear antigen (PCNA) in prostatic carcinomas to various clinical parameters. Prostate. 1992; 20:243–53. 10.1002/pros.2990200309. 1374182

[31] Mulligan JM , Mai KT , Parks W , Gerridzen RG . Proliferating cell nuclear antigen (PCNA) and MIB 1: Markers of locally advanced and biologically aggressive prostate cancer. Can J Urol. 1997; 4:422–25. 12735823

[32] Stuart-Harris R , Caldas C , Pinder SE , Pharoah P . Proliferation markers and survival in early breast cancer: a systematic review and meta-analysis of 85 studies in 32,825 patients. Breast. 2008; 17:323–34. 10.1016/j.breast.2008.02.002. 18455396

[33] Kimos MC , Wang S , Borkowski A , Yang GY , Yang CS , Perry K , Olaru A , Deacu E , Sterian A , Cottrell J , Papadimitriou J , Sisodia L , Selaru FM , et al. Esophagin and proliferating cell nuclear antigen (PCNA) are biomarkers of human esophageal neoplastic progression. Int J Cancer. 2004; 111:415–17. 10.1002/ijc.20267. 15221970

[34] Lemech CR , Kichenadasse G , Marschner JP , Alevizopoulos K , Otterlei M , Millward M . ATX-101, a cell-penetrating protein targeting PCNA, can be safely administered as intravenous infusion in patients and shows clinical activity in a Phase 1 study. Oncogene. 2023; 42:541–44. 10.1038/s41388-022-02582-6. 36564469 PMC9918429

[35] Tan Z , Wortman M , Dillehay KL , Seibel WL , Evelyn CR , Smith SJ , Malkas LH , Zheng Y , Lu S , Dong Z . Small-molecule targeting of proliferating cell nuclear antigen chromatin association inhibits tumor cell growth. Mol Pharmacol. 2012; 81:811–19. 10.1124/mol.112.077735. 22399488 PMC3362894

[36] Dillehay KL , Seibel WL , Zhao D , Lu S , Dong Z . Target validation and structure-activity analysis of a series of novel PCNA inhibitors. Pharmacol Res Perspect. 2015; 3:e00115. 10.1002/prp2.115. 25729582 PMC4324689

[37] Lu S , Dong Z . Additive effects of a small molecular PCNA inhibitor PCNA-I1S and DNA damaging agents on growth inhibition and DNA damage in prostate and lung cancer cells. PLoS One. 2019; 14:e0223894. 10.1371/journal.pone.0223894. 31600334 PMC6786632

[38] Dillehay KL , Lu S , Dong Z . Antitumor effects of a novel small molecule targeting PCNA chromatin association in prostate cancer. Mol Cancer Ther. 2014; 13:2817–26. 10.1158/1535-7163.MCT-14-0522. 25253786

[39] Lu S , Dong Z . Proliferating cell nuclear antigen directly interacts with androgen receptor and enhances androgen receptor-mediated signaling. Int J Oncol. 2021; 59:41. 10.3892/ijo.2021.5221. 33982774 PMC8131087

[40] Dai JL , Maiorino CA , Gkonos PJ , Burnstein KL . Androgenic up-regulation of androgen receptor cDNA expression in androgen-independent prostate cancer cells. Steroids. 1996; 61:531–39. 10.1016/s0039-128x(96)00086-4. 8883219

[41] Jones D , Wade M , Nakjang S , Chaytor L , Grey J , Robson CN , Gaughan L . FOXA1 regulates androgen receptor variant activity in models of castrate-resistant prostate cancer. Oncotarget. 2015; 6:29782–94. 10.18632/oncotarget.4927. 26336819 PMC4745762

[42] Kounatidou E , Nakjang S , McCracken SRC , Dehm SM , Robson CN , Jones D , Gaughan L . A novel CRISPR-engineered prostate cancer cell line defines the AR-V transcriptome and identifies PARP inhibitor sensitivities. Nucleic Acids Res. 2019; 47:5634–47. 10.1093/nar/gkz286. 31006810 PMC6582326

[43] Hu R , Lu C , Mostaghel EA , Yegnasubramanian S , Gurel M , Tannahill C , Edwards J , Isaacs WB , Nelson PS , Bluemn E , Plymate SR , Luo J . Distinct transcriptional programs mediated by the ligand-dependent full-length androgen receptor and its splice variants in castration-resistant prostate cancer. Cancer Res. 2012; 72:3457–62. 10.1158/0008-5472.CAN-11-3892. 22710436 PMC3415705

[44] van de Wijngaart DJ , Dubbink HJ , van Royen ME , Trapman J , Jenster G . Androgen receptor coregulators: recruitment via the coactivator binding groove. Mol Cell Endocrinol. 2012; 352:57–69. 10.1016/j.mce.2011.08.007. 21871527

[45] Jin F , Fondell JD . A novel androgen receptor-binding element modulates Cdc6 transcription in prostate cancer cells during cell-cycle progression. Nucleic Acids Res. 2009; 37:4826–38. 10.1093/nar/gkp510. 19520769 PMC2724301

[46] Hörnberg E , Ylitalo EB , Crnalic S , Antti H , Stattin P , Widmark A , Bergh A , Wikström P . Expression of androgen receptor splice variants in prostate cancer bone metastases is associated with castration-resistance and short survival. PLoS One. 2011; 6:e19059. 10.1371/journal.pone.0019059. 21552559 PMC3084247

[47] Hochegger H , Takeda S , Hunt T . Cyclin-dependent kinases and cell-cycle transitions: does one fit all? Nat Rev Mol Cell Biol. 2008; 9:910–16. 10.1038/nrm2510. 18813291

[48] Gu P , Yang D , Zhu J , Zhang M , He X . Bioinformatics analysis identified hub genes in prostate cancer tumorigenesis and metastasis. Math Biosci Eng. 2021; 18:3180–96. 10.3934/mbe.2021158. 34198380

[49] Yang R , Du Y , Wang L , Chen Z , Liu X . Weighted gene co-expression network analysis identifies CCNA2 as a treatment target of prostate cancer through inhibiting cell cycle. J Cancer. 2020; 11:1203–11. 10.7150/jca.38173. 31956366 PMC6959059

[50] Liu K , Chen Y , Feng P , Wang Y , Sun M , Song T , Tan J , Li C , Liu S , Kong Q , Zhang J . Identification of Pathologic and Prognostic Genes in Prostate Cancer Based on Database Mining. Front Genet. 2022; 13:854531. 10.3389/fgene.2022.854531. 35360870 PMC8963346

[51] Wang Z , Zou J , Zhang L , Liu H , Jiang B , Liang Y , Zhang Y . Comprehensive analysis of the progression mechanisms of CRPC and its inhibitor discovery based on machine learning algorithms. Front Genet. 2023; 14:1184704. 10.3389/fgene.2023.1184704. 37476415 PMC10354439

[52] Liu M , Zhang Y , Zhang A , Deng Y , Gao X , Wang J , Wang Y , Wang S , Liu J , Chen S , Yao W , Liu X . Compound K is a potential clinical anticancer agent in prostate cancer by arresting cell cycle. Phytomedicine. 2023; 109:154584. 10.1016/j.phymed.2022.154584. 36610114

[53] Sharp A , Coleman I , Yuan W , Sprenger C , Dolling D , Rodrigues DN , Russo JW , Figueiredo I , Bertan C , Seed G , Riisnaes R , Uo T , Neeb A , et al. Androgen receptor splice variant-7 expression emerges with castration resistance in prostate cancer. J Clin Invest. 2019; 129:192–208. 10.1172/JCI122819. 30334814 PMC6307949

[54] Lu S , Lamba M , Wang J , Dong Z . Targeting proliferating cell nuclear antigen enhances ionizing radiation-induced cytotoxicity in prostate cancer cells. Prostate. 2024; 84:1456–67. 10.1002/pros.24786. 39219052

[55] Jamshidi M , Keshavarzi F , Amini S , Laher I , Gheysarzadeh A , Davari K . Targeting androgen receptor (AR) with a synthetic peptide increases apoptosis in triple negative breast cancer and AR-expressing prostate cancer cell lines. Cancer Rep (Hoboken). 2024; 7:e1922. 10.1002/cnr2.1922. 37903548 PMC10809188

[56] Pilling AB , Hwang O , Boudreault A , Laurent A , Hwang C . IAP Antagonists Enhance Apoptotic Response to Enzalutamide in Castration-Resistant Prostate Cancer Cells via Autocrine TNF-α Signaling. Prostate. 2017; 77:866–77. 10.1002/pros.23327. 28240376

[57] Lu S , Liu M , Epner DE , Tsai SY , Tsai MJ . Androgen regulation of the cyclin-dependent kinase inhibitor p21 gene through an androgen response element in the proximal promoter. Mol Endocrinol. 1999; 13:376–84. 10.1210/mend.13.3.0254. 10076995

[58] Lu S , Jenster G , Epner DE . Androgen induction of cyclin-dependent kinase inhibitor p21 gene: role of androgen receptor and transcription factor Sp1 complex. Mol Endocrinol. 2000; 14:753–60. 10.1210/mend.14.5.0461. 10809237

[59] Nyquist MD , Li Y , Hwang TH , Manlove LS , Vessella RL , Silverstein KA , Voytas DF , Dehm SM . TALEN-engineered AR gene rearrangements reveal endocrine uncoupling of androgen receptor in prostate cancer. Proc Natl Acad Sci U S A. 2013; 110:17492–97. 10.1073/pnas.1308587110. 24101480 PMC3808622

[60] Dong Z , Liu Y , Scott KF , Levin L , Gaitonde K , Bracken RB , Burke B , Zhai QJ , Wang J , Oleksowicz L , Lu S . Secretory phospholipase A2-IIa is involved in prostate cancer progression and may potentially serve as a biomarker for prostate cancer. Carcinogenesis. 2010; 31:1948–55. 10.1093/carcin/bgq188. 20837598 PMC2981059

[61] Sharma A , Yeow WS , Ertel A , Coleman I , Clegg N , Thangavel C , Morrissey C , Zhang X , Comstock CE , Witkiewicz AK , Gomella L , Knudsen ES , Nelson PS , Knudsen KE . The retinoblastoma tumor suppressor controls androgen signaling and human prostate cancer progression. J Clin Invest. 2010; 120:4478–92. 10.1172/JCI44239. 21099110 PMC2993601

